# Visible-Light-Induced Diselenide-Crosslinked Polymeric Micelles for ROS-Triggered Drug Delivery

**DOI:** 10.3390/molecules29163970

**Published:** 2024-08-22

**Authors:** Xinfeng Cheng, Huixian Li, Xiaomeng Sun, Tianxu Xu, Zhenzhen Guo, Xianchao Du, Shuai Li, Xuyang Li, Xiaojing Xing, Dongfang Qiu

**Affiliations:** College of Chemistry and Pharmaceutical Engineering, Nanyang Normal University, Nanyang 473061, China

**Keywords:** visible-light sensitivity, diselenide, crosslinking, ROS responsiveness, drug delivery

## Abstract

To synthesize an effective and versatile nano-platform serving as a promising carrier for controlled drug delivery, visible-light-induced diselenide-crosslinked polyurethane micelles were designed and prepared for ROS-triggered on-demand doxorubicin (DOX) release. A rationally designed amphiphilic block copolymer, poly(ethylene glycol)-*b*-poly(diselenolane diol-co-isophorone diisocyanate)-*b*-poly(ethylene glycol) (PEG-*b*-PUSe-*b*-PEG), which incorporates dangling diselenolane groups within the hydrophobic PU segments, was initially synthesized through the polycondensation reaction. In aqueous media, this type of amphiphilic block copolymer can self-assemble into micellar aggregates and encapsulate DOX within the micellar core, forming DOX-loaded micelles that are subsequently in situ core-crosslinked by diselenides via a visible-light-triggered metathesis reaction of Se-Se bonds. Compared with the non-crosslinked micelles (NCLMs), the as-prepared diselenide-crosslinked micelles (CLMs) exhibited a smaller particle size and improved colloidal stability. In vitro release studies have demonstrated suppressed drug release behavior for CLMs in physiological conditions, as compared to the NCLMs, whereas a burst release of DOX occurred upon exposure to an oxidation environment. Moreover, MTT assay results have revealed that the crosslinked polyurethane micelles displayed no significant cytotoxicity towards HeLa cells. Cellular uptake analyses have suggested the effective internalization of DOX-loaded crosslinked micelles and DOX release within cancer cells. These findings suggest that this kind of ROS-triggered reversibly crosslinked polyurethane micelles hold significant potential as a ROS-responsive drug delivery system.

## 1. Introduction

Polymeric micelles that respond to various stimuli have gained recognition as promising smart nanocarriers for the precise and controlled delivery of therapeutics. Nevertheless, most polymer micelles suffer from low structural stability, premature drug release at undesired sites, insufficient intracellular drug delivery, etc. [1,2,3]. Recently, a crosslinking strategy has been found to be an efficient methodology to prepare many kinds of crosslinked polymeric nanoparticles with enhanced structural stability [4,5,6,7]. However, the excessive stability enabled by crosslinking can prevent crosslinked micelles from the timely and effective release of entrapped drugs at target sites. In order to balance these competing issues, the reversible crosslinking of polymeric micelles via stimuli-triggered cleavable linkages provides an effective strategy to overcome these challenges [8,9]. In recent years, there have been a range of shell, core, and shell–core interface crosslinked micellar nanocarriers via various cleavable crosslinkers found, including acid-labile imine and acetal, redox-labile disulfide, photo-cleavable coumarin, thermally labile Diels–Alder, and Azo linkages [10,11,12,13,14,15]. Among them, disulfide is an extraordinary promising candidate for bio-responsive reversibly crosslinked micellar nanocarriers, owing to its site-specific decrosslinking and controlled drug delivery in tumor tissues with an elevated reductive glutathione GSH level (0.5–10 mM) relative to normal tissues (2–20 µM) [16,17]. Therefore, a variety of redox-responsive reversibly crosslinked micelles based on disulfides have been extensively engineered for targeted tumor drug delivery [18,19,20,21].

Besides disulfide bonds, more and more efforts have focused on diselenide-containing polymers in recent years to pursue their appropriate application in controlled drug delivery systems like diselenide-containing counterparts [22,23,24,25,26,27,28]. The energy of the Se-Se bond (172 kJ mol^−1^) is less than that of the S-S bond (240 kJ mol^−1^). Owing to this excellent stimulus sensitivity, diselenide bonds can be easily cleaved under milder redox conditions [29]. Recently, diselenide bonds have been extensively investigated and utilized as reversible crosslinkers incorporated into micelles for GSH- and reactive oxygen species (ROS)-induced on-demand drug delivery [30,31,32,33,34,35,36,37]. In this regard, Park et al. developed in situ diselenide-crosslinked polymeric micelles containing PEG and polypeptide building blocks via an active selenol self-coupling reaction to achieve improved structural stability and site-specific drug release [30]. Lim and coworkers developed an indocyanine green (ICG)-encapsulated near-infrared-light-induced diselenide-core-crosslinked micellar nanocarrier via the post-crosslinking method (i.e., Diels–Alder click reaction) using a diselenide-containing bismaleimide crosslinker [31]. Although there have been several studies conducted on the preparation of redox-responsive diselenide-crosslinked micellar nanocarriers via the generally used crosslinking strategy, it is also highly desirable to develop an innovative facile approach to fabricate feasible and efficient diselenide-stabilized micellar aggregates for site-specific on-demand drug delivery. For instance, enlightened by the visible-light-induced diselenide metathesis reaction proposed by Xu and coworkers [38], Xing et al. prepared a kind of in situ core-crosslinked micelle (PEG-*b*-PBSe) via diselenides with enhanced physiological stability for efficient redox-responsive dual drug delivery [32]. This visible-light-mediated photo-crosslinking method provides a mild and efficient strategy for the development of reversibly crosslinked micellar nano-delivery systems based on diselenides [33,34]. Such an approach is expected to possess multiple desired features, such as convenient synthesis under mild conditions, in situ formation, no crosslinking agent or other reagent addition, no generation of by-products, and no further purification requirement during the procedure, which greatly inspired our design ideas for novel diselenide-based reversibly crosslinked polymeric micelle systems.

**Scheme 1:**
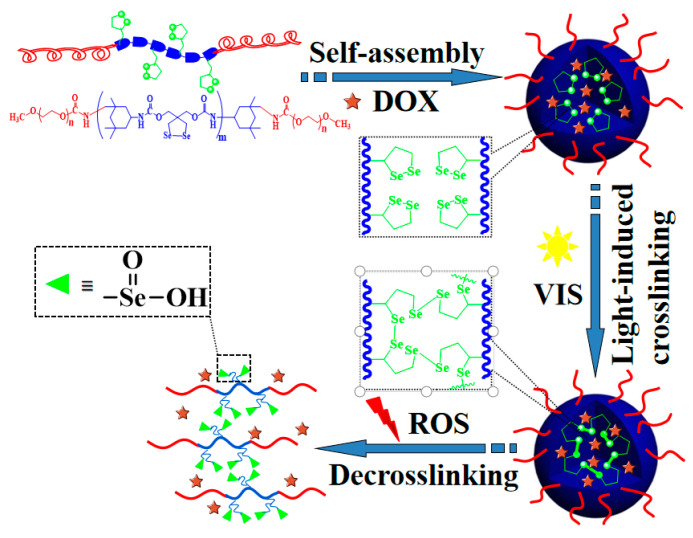
Representation of the formation of diselenide-crosslinked MPEG-*b*-PUSe-*b*-MPEG micelles and their ROS-responsive drug release.

Herein, a novel kind of diselenide-crosslinked polymeric micelle was rationally designed by the core-crosslinking of diselenolane-containing PEGylated polyurethane (PU) micelles via a visible-light-induced Se-Se exchange reaction, as shown in Scheme 1. To our knowledge, this is the first presentation of adopting this form of polyurethane bearing diselenolane groups for the convenient and efficient preparation of diselenide-crosslinked micelles. The amphiphilic block copolymers MPEG-*b*-PUSe-*b*-MPEG bearing dangling diselenolane groups were designed and synthesized utilizing a typical polycondensation reaction of diselenolane diol and isophorone diisocyanate (IPDI), and then terminated using methoxy poly(ethylene glycol) (MPEG). They could form micellar aggregates in an aqueous solution via self-assembly behavior and could be further crosslinked by diselenide linkages via visible-light-induced Se-Se exchange reaction. The self-assembly behavior and structural stability of the polymeric micelles before and after the visible light irradiation were examined using DLS, TEM, and UV-Vis spectra analyses. Moreover, the stimuli responsiveness and in vitro drug release patterns of the DOX-loaded diselenide-crosslinked micelles were also investigated in detail. This kind of micelle was found to possess enhanced structural stability, minimal drug leakage when treated with mild visible light, and underwent ROS-triggered disruption and on-demand drug release behaviors, exhibiting immense promise as a regulated drug delivery system. Thus, this exploratory study provides a novel and convenient method for the preparation of reversibly crosslinked micelles with desirable colloidal stability and controlled release characteristics.

## 2. Results and Discussion

### 2.1. Synthesis and Characterization

**Scheme 2:**
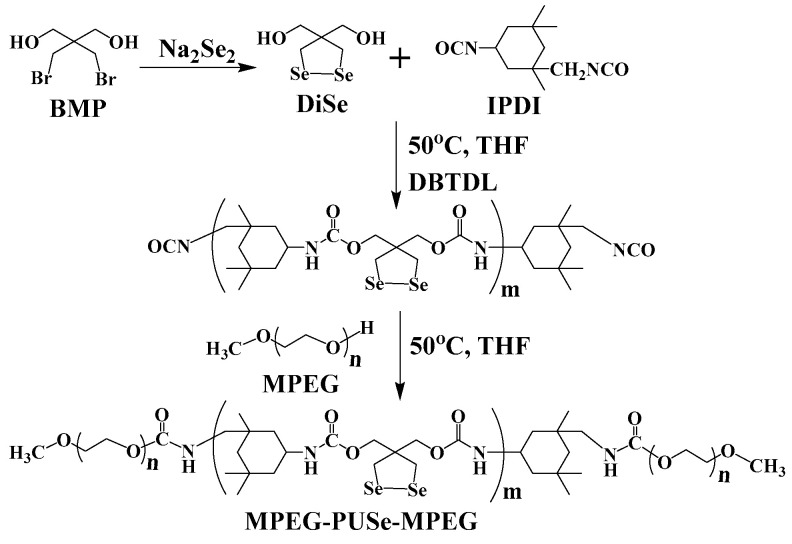
The synthetic route of the monomer DiSe and MPEG-*b*-PUSe-*b*-MPEG triblock copolymers.

**Figure 1:**
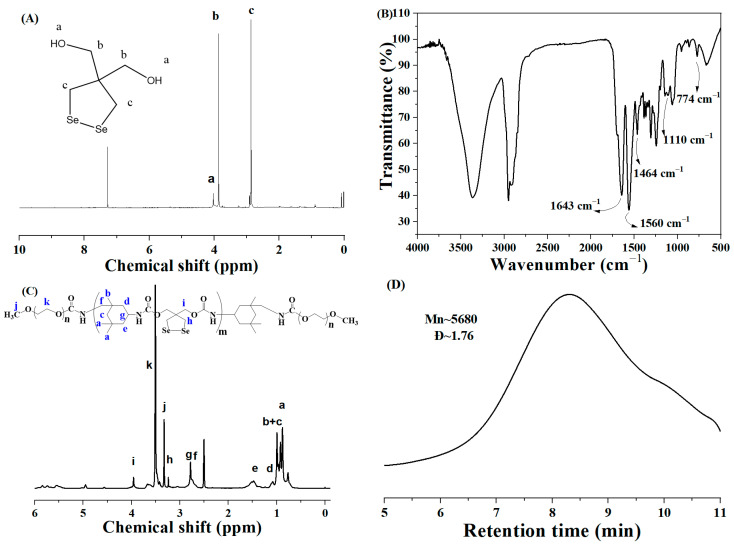
(**A**) The ^1^H NMR spectrum of the functional monomer DiSe; (**B**–**D**) FT-IR, ^1^H NMR spectra, and GPC trace of MPEG-*b*-PUSe-*b*-MPEG copolymers, respectively. (Letters are used solely to clearly indicate the corresponding proton po-sitions in the product structure as represented by the various proton peaks in the NMR spectrum.)

To develop a novel easy approach to fabricate feasible and efficient diselenide-stabilized micellar nanocarriers for on-demand drug delivery, a rationally designed triblock copolymer MPEG-*b*-PUSe-*b*-MPEG bearing functional diselenolane groups was easily synthesized, as shown in Scheme 2. A functional monomer diselenolane diol (DiSe) containing a cyclic diselenide group was initially synthesized and verified by structural analysis. From the ^1^H NMR spectrum (Figure 1A), the characteristic diselenolane and hydroxymethyl proton signals located at 2.86 and 3.87 ppm, respectively, proved the formation of functional cyclic diselenide structure. This was further confirmed by the ^13^C NMR spectrum (Figure S1) with characteristic signals at 14.9 (SeCH_2_-), 49.0 (C(CH_2_OH)_2_), and 68.9 ppm (CH_2_OH).

The triblock copolymers MPEG-*b*-PUSe-*b*-MPEG were easily synthesized via the polycondensation reaction of DiSe with IPDI to give the prepolymers PUSe. Then, they were terminated utilizing MPEG blocks to give the final copolymers. They were then examined using FT-IR, NMR, and GPC analyses. Compared to the active –NCO groups terminated prepolymer PUSe (Figure S2), it is obvious that no characteristic band around 2270 cm^−1^ belonging to –NCO groups was found in Figure 1B. This indicates the complete consumption of introduced IPDI units. These characteristic vibrations, together with other bands located at 1643 and 1560 (–NHCOO–), 1110 (C–O–C), and 774 (C–Se) cm^−1^, revealed the coexistence of IPDI, DiSe, and MPEG segments along the copolymer backbones. Besides, the ^1^H NMR spectra of the prepolymers PUSe (Figure S2) and the copolymers MPEG-*b*-PUSe-*b*-MPEG (Figure 1C) were also characterized. In the ^1^H NMR spectrum of MPEG-*b*-PUSe-*b*-MPEG, the proton signals of IPDI units ranged from δ 0.85 to 1.52, signals belonging to DiSe units appeared at δ 3.24 (-C*H*_2_SeSeC*H*_2_-) and 3.95 (-O-C*H*_2_-C-C*H*_2_-O-), and a strong signal peak around δ 3.50 corresponded to the -C*H*_2_C*H*_2_O- of MPEG segments. The molecular weight of the copolymer estimated from the ^1^H NMR spectrum result was determined as 5190 by comparing the proton peak integrals of the diselenolane ring of DiSe with ethoxy moieties of MPEG. Moreover, the number average molecular weight (M_n_) of the resulting copolymer as determined by GPC measurement was 5680 with a dispersity of 1.76 (Figure 1D). This was close to the value determined by the NMR analysis. Thus, the aforementioned analyses confirmed that the MPEG-*b*-PUSe-*b*-MPEG copolymer possessing a well-defined structure was successfully synthesized.

### 2.2. Self-Assembly Behavior of MPEG-b-PUSe-b-MPEG Block Copolymers

The as-prepared block copolymers tend to form micellar aggregates through self-assembly behavior in an aqueous solution above a critical concentration. This is because of the amphiphilic characteristic of the as-prepared block copolymers. The as-prepared triblock MPEG-*b*-PUSe-*b*-MPEG copolymers demonstrated a self-assembled core–shell micellar structure. This included inner PU hydrophobic cores and outer, MPEG hydrophilic shells, as illustrated in Scheme 1. A typical fluorescence molecule pyrene was chosen as a probe for identifying the critical micelle concentration (CMC) of the MPEG-*b*-PUSe-*b*-MPEG copolymers to verify this self-assembly behavior [39]. It is known that the fluorescence intensity of the pyrene is sensitive to the polarity of its surrounding microenvironment. The fluorescence intensities of pyrene in different concentrations of copolymer aqueous solutions were monitored utilizing a PerkinElmer LS50 fluorescence spectrometer. The characteristic intensity ratio I_384_/I_373_ of pyrene was used to determine the CMC values of MPEG-*b*-PUSe-*b*-MPEG in relation to the logarithmic concentrations of the copolymers (Figure S3). The CMC value of the MPEG-*b*-PUSe-*b*-MPEG copolymers determined from the intersection was 6.0 ± 1.5×10^−3^ mg/mL (~1.06 μM), which was comparable to that of other related amphiphilic polyurethane copolymers with similar structures [40,41]. This lower CMC value may enable the maintenance of micellar dynamic structure against dilution in blood circulation.

**Figure 2:**
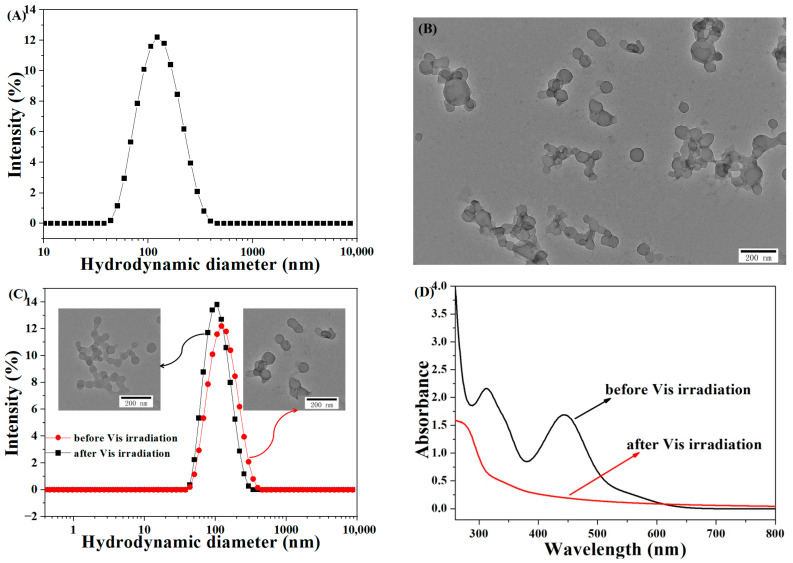
(**A**) DLS data and (**B**) the TEM image of the MPEG-*b*-PUSe-*b*-MPEG micelle solution (micelle concentration: 0.5 mg/mL); (**C**) the DLS data (inset: TEM images) of the MPEG-*b*-PUSe-*b*-MPEG micelles before or after Vis irradiation (micelle concentration: 0.5 mg/mL); (**D**) the UV-Vis spectra of the MPEG-*b*-PUSe-*b*-MPEG micelles before or after Vis irradiation.

The corresponding MPEG-*b*-PUSe-*b*-MPEG micelles were prepared using a dissolution and dialysis approach, which were characterized by their size and morphology using DLS and TEM analyses. The DLS analysis showed an average micelle diameter of 102 ± 5 nm and a polydispersity index (PDI) of 0.169 ± 0.008 (Figure 2A). The TEM tests demonstrated a typical spherical structure with a mean diameter of 95 ± 9 nm (Figure 2B). The difference in the size of the MPEG-*b*-PUSe-*b*-MPEG micellar aggregates tested using DLS and TEM was ascribed to hydrated and dehydrated states in the DLS and TEM samples, respectively.

### 2.3. Visible-Light-Induced Diselenide-Crosslinked MPEG-b-PUSe-b-MPEG Micelles

As an emerging building unit, diselenide bonds possessing redox dual sensitivity and dynamic covalent characteristics have been incorporated into various polymer architectures. These diselenide-based polymer systems have attracted growing attention as biomaterials and dynamic materials, including nanocarriers, hydrogels, and reverse materials. Particularly, by virtue of their exciting exchange reaction under mild visible light, these bonds have been widely explored and applied potentially in diverse types of smart materials, developed as interesting smart materials including self-healing, photo-patterning, and surface-modifying materials [42,43,44,45]. However, this visible-light-induced dynamic diselenide chemistry has seldom been adopted as a mild and efficient strategy to tune the stability of the nanomicelle systems via photo-crosslinking. Considering the multi-advantages of the photo-crosslinking method, such as in situ and mild synthesis, no crosslinker or extra reagent addition, no by-product generation, and no further purification requirement during the procedure, the visible-light-induced photo-crosslinking method is expected to be an effective approach for modulating the stability of polymeric micelles via dynamic diselenide exchange.

Thus, in this study, the visible-light-induced Se-Se exchange reaction was adopted to prepare diselenide-crosslinked micelle systems. Upon irradiation by visible light, the variation in particle size and morphology of the micelles was examined using the DLS and TEM techniques. The DLS data indicated a reduction in the micelle size of 90 ± 6 nm and a PDI of 0.147 ± 0.005 after visible light irradiation (Figure 2C). Apparently, the corresponding TEM observations exhibited a spherical structure with a smaller size relative to the non-irradiated ones. The size differences before and after the visible light irradiation may be attributed to the photo-crosslinking behavior of the precursor micelles with pendent dynamic diselenide groups, forming a more compact structure under irradiation [46,47]. Notably, as is evident from the UV-Vis spectra in Figure 2D, a distinct characteristic peak existed at 443 nm for the precursor micelles before the irradiation belonging to the unique diselenolane structure [48]. However, this characteristic absorption vanished after visible light irradiation, resulting from the ring-opening exchange reaction of cyclic diselenolane groups. This is similar to that of dithiolane rings containing micellar particles when treated by catalytic dithiothreitol (DTT) or dialysis procedure [49].

Furthermore, a small-molecule model compound, i.e., DiSe, was chosen to verify this ring-opening reaction under visible light. It was exciting to see that the characteristic signals of DiSe molecules located at 2.86 and 3.87 ppm were significantly broadened, corresponding to a typical signature of the oligomers or polymers (Figure S4). In view of these interesting observations, it seemed reasonable to infer that the cyclic diselenolane rings experienced a photo-induced ring-opening polymerization to generate the linear oligo(dielenides) and poly(dielenides) under visible light irradiation. Given the exciting findings outlined above, this photo-induced ring-opening crosslinking method turned out to be a convenient and efficient strategy for preparing reversibly crosslinked micelles without any catalyst addition and by-product generation.

### 2.4. The Structural Stability of the Micelles

**Figure 3:**
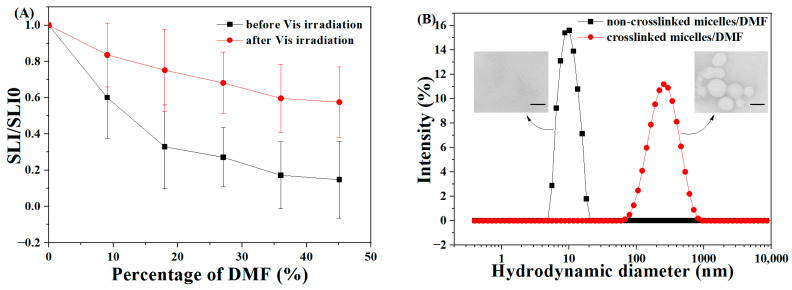
(**A**) Variation in relative scattered light intensity (SLI/SLI_0_) upon dilution by different volumes of DMF for MPEG-*b*-PUSe-*b*-MPEG micelles in the aqueous solution (initial concentration: 0.5 mg/mL) before and after Vis-irradiation. Data are shown as mean ± SD (*n* = 3). (**B**) The DLS data and TEM images of lyophilized non-crosslinked and crosslinked micelles in DMF solution (Scale bar: 200 nm).

The structural stability of the as-prepared micelles was tested in severe conditions. As a well-known good solvent for dissolving the as-prepared amphiphilic block polyurethanes, DMF was chosen as a dilution solvent to investigate the structural stability of the micelles [46,50]. The gradient dilution of the resulting micelles before or after the crosslinking was performed utilizing DMF solvent at volume ratios of 10~45%; the corresponding scattering light intensities (SLI) were detected in real-time using DLS. As demonstrated in Figure 3A, it is apparent from the SLI data that the non-crosslinked micelles (NCLMs) showed a significant decrease upon dilution, accompanied by lower retention (~14%) of its initial SLI relative to the diselenide-crosslinked ones (~57%). These observations might be attributed to the visible-light-induced crosslinking structure, which seemed to strengthen the thermodynamic stability of the micelles against the extreme environment. The diselenide-crosslinked micelles (CLMs) also displayed enhanced stability against dilution in simulated physiological conditions. The diselenide-crosslinked micelles exhibited only a slight increase (114 ± 5 nm) in size after being diluted by 100-fold pH 7.4 PBS solution. In contrast, the NCLMs displayed distinct dissociation behavior to form small aggregates of approximately 13 ± 7 nm upon dilution (Figure S5).

Besides, the lyophilized samples of the non-crosslinked and crosslinked micelles redispersed in pure organic solvent DMF were also studied for comparison. The corresponding colloidal properties of the above micelles including the particle size, distribution, and morphology were examined using DLS and TEM. As is obvious from the DLS data depicted in Figure 3B, the average diameter of the crosslinked micelles in DMF increased to 297 ± 10 nm along with a PDI of 0.336 ± 0.011. In contrast, the non-crosslinked ones displayed a dramatic decrease down to about 10 nm compared to their counterparts in water. The corresponding TEM results showed that no typical aggregates were detected, and only a few unimers remained on the substrate for the NCLMs. There were some spherical particles with bigger sizes ranging from 110 to 230 nm for the CLMs (inset of Figure 3B). The increased micellar size of the crosslinked micelles in the DMF solution compared to the aqueous solution was ascribed to the swelling of the crosslinked PU cores in DMF. Moreover, due to the good dissolution characteristics of DMF for the as-prepared amphiphilic block polyurethanes, no aggregates were found when dispersing the NCLMs in DMF. Given the above findings, the visible-light-induced diselenide-crosslinking method could be an efficient way to achieve micelles with enhanced structural stability against dilution.

### 2.5. ROS-Induced Disassembly Behavior of Diselenide-Crosslinked Micelles (CLMs)

**Figure 4:**
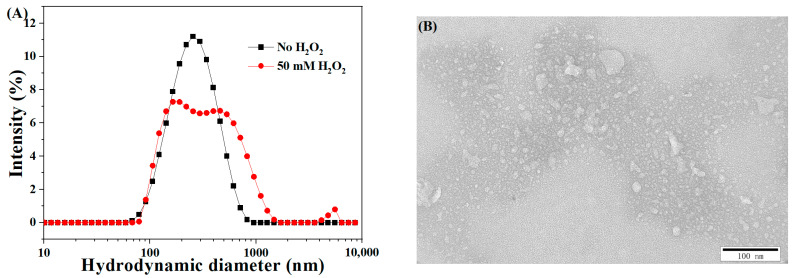
The size variation (**A**) and TEM image (**B**) of the CLMs upon exposure to 50 mM H_2_O_2_ (micelle concentration: 0.5 mg/mL).

The ROS-induced disassembly behavior of CLMs was investigated via the DLS and TEM analyses. As illustrated in Figure 4A, there were pronounced size distribution changes in CLMs upon oxidative stress. The size of the crosslinked micelles was broadened to be above 1 μm with a distribution of 0.485 when exposed to 50 mM H_2_O_2_. This broadened size variation in CLMs may be possibly due to the oxidation-triggered diselenide crosslink cleavage, resulting in the formation of more hydrophilic seleninic acid groups in the micellar cores (Scheme 1) [29]. Combined with the contribution of the hydrophilic PEG shell, the entire micelles became more hydrophilic and tended to swell, dissolve, and disassemble into water-soluble polymer fragments or larger-size polymer clusters. Additionally, as shown in Figure 4B, the TEM images of the CLMs depicted unstructured or large aggregates triggered by oxidation stimulus, further confirming the aforementioned ROS-induced disassembly behavior of the CLMs.

### 2.6. DSC Analysis

**Figure 5:**
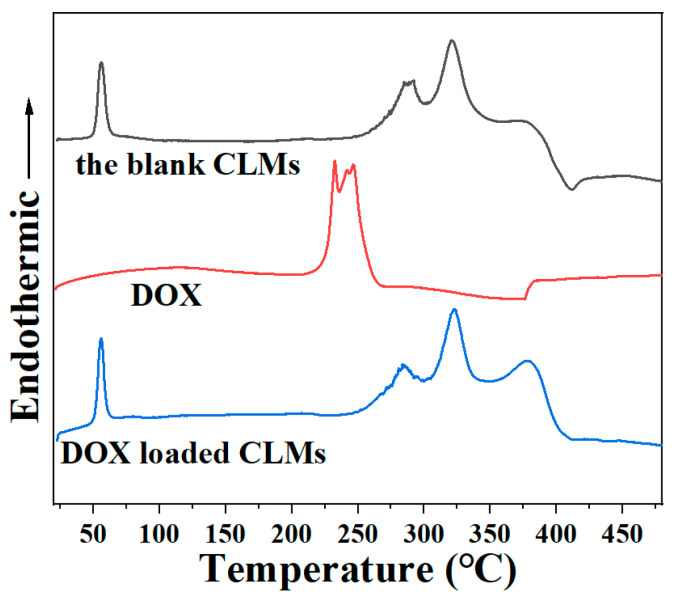
DSC thermograms of the blank CLMs, DOX, and DOX-loaded CLMs.

The thermal performances of the blank CLMs, as well as DOX and DOX-loaded CLMs, were examined. As illustrated in Figure 5, the lyophilized blank CLMs exhibited an endothermic peak at 56.19 °C, reflecting the melting of the crystalline PEG segments. There were also three definite endothermic peaks at 283.52, 320.13, and 376.31 °C, corresponding to the characteristic thermal decomposition of the hard and soft segments of the MPEG-*b*-PUSe-*b*-MPEG polyurethane copolymers. The endothermic peak of DOX at 232.10 and 246.35 °C indicated its thermal stability. For the lyophilized DOX-loaded CLMs, there were no characteristic melting peaks of DOX, but the endothermic peaks of MPEG-*b*-PUSe-*b*-MPEG copolymers were approximately at the same position as the blank CLMs. These results indicate a reduction in the crystallinity of DOX within the formulation and suggest potential interactions between the MPEG-*b*-PUSe-*b*-MPEG copolymer and DOX, which may enhance the loading efficiency and bioavailability of the entrapped DOX. 

### 2.7. In Vitro Drug Release Behavior of DOX-Loaded Micelles

DOX, a typical anticancer drug, was chosen as a model to assess the drug release characteristics of the micelles. The drug loading content (DLC) and drug loading efficiency (DLE) of the as-prepared CLMs were determined to be 20.7% ± 1.9% and 72.6% ± 4.6%, respectively. However, for the NCLMs, the values somehow decreased (DLC~13.2% ± 1.25%; DLE~49.5% ± 3.4%) compared to the diselenide-crosslinked ones. This difference may be attributed to the more compact inner cores of the CLMs, which could efficiently prevent DOX leakage during the following dialysis procedure. In addition, the average diameters of the DOX-loaded crosslinked micelles (CLMs) and NCLMs were 152 ± 3.5 and 178 ± 5.6 nm, respectively (Figure S6). The size of DOX-loaded NCLMs was a little larger relative to the blank micelles, possibly due to the hydrophobic interaction of DOX with the inner PU cores of the micelles.

**Figure 6:**
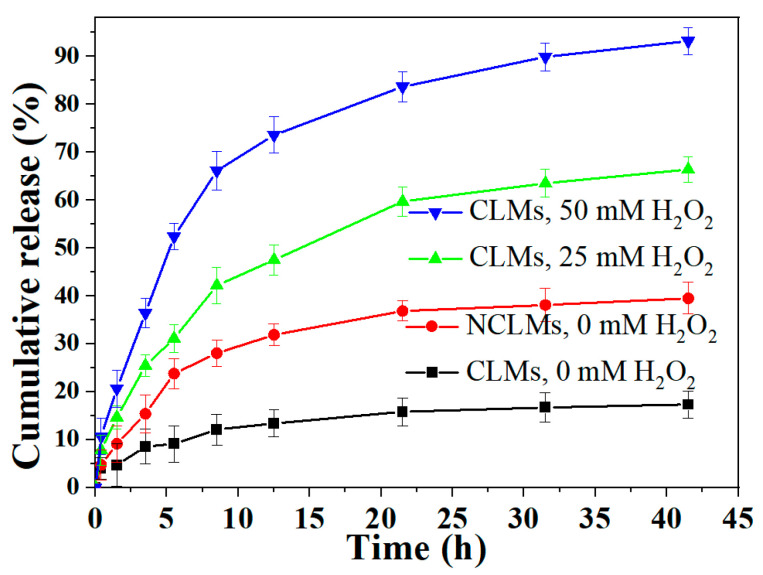
In vitro DOX release profiles of DOX-loaded NCLMs and CLMs with or without H_2_O_2_ at pH 7.4 (micelle concentration: 1 mg/mL). Data are shown as mean ± SD (*n* = 3).

Different buffered solutions with varying oxidant (H_2_O_2_) concentrations were chosen as release media to investigate the drug release behavior of the drug-loaded CLMs. As depicted in Figure 6, only about 17% ± 3.1% of the drug DOX was released gradually over a 30 h period without oxidation stimulus for the DOX-loaded CLMs. In contrast, the NCLMs displayed a slightly faster release rate of 38% ± 3.5% within 30 h. The slower release rate in the former case was attributed to the effective crosslinking of the inner cores of the CLMs, which could prevent the premature release of DOX from the micelles. Additionally, when incubated in the pH 7.4 PBS solution for 40 h, DLS measurements revealed that the size of the DOX-loaded CLMs exhibited only a slight increase (173 ± 4.6 nm). However, the DOX-loaded NCLMs exhibited a significant increase in size (294 ± 10.4 nm). This further reveals the enhanced structural stability of the DOX-loaded CLMs against swelling in an imitated physiological environment (Figure S6). Thus, the premature release of the encapsulated DOX drugs from these CLMs could be effectively prevented. Upon adding an oxidative stimulus (25 mM H_2_O_2_), the release rate of DOX increased significantly, resulting in about 66% ± 2.7% of drug release within 40 h. This enhancement occurred as the oxidative stimulus triggered the breakage of Se-Se bonds, leading to the rapid disassembly of CLMs and accelerated DOX release. Notably, when the environmental oxidant (H_2_O_2_) concentration was elevated to 50 mM, DOX exhibited an even faster release behavior and nearly a complete release (93% ± 2.8% in 40 h) from the micelles. From the above observations, it can be seen that DOX drugs could be efficiently protected by the diselenide-crosslinked micelles, and controlled release could be triggered by oxidative conditions. It suggests that the micelles crosslinked with diselenides could serve as efficient drug carriers.

Moreover, the following semiempirical model proposed by Ritger and Peppas was used to fit the accumulative release profiles to understand the release mechanism of DOX payloads from the diselenide-crosslinked micelles [51,52]:

*M_t_*/*M_∞_* = *kt^n^*   (for *M_t_*/*M_∞_* ≤ 0.6)

where *M_t_* represents the cumulative release of the encapsulated drugs at a specific time, while *M_∞_* signifies the total release amount as time approaches infinity. The variable *k* refers to the rate constant. The drug release mechanism can be elucidated from the release exponent *n*. When the *n* value is 0.5, 1, 0.5 < *n* < 1, and *n* < 0.5, the drug release mechanism corresponds to Fickian diffusion, zero-order release, anomalous transport, and pseudo-Fickian diffusion, respectively.

The release kinetic parameters obtained by fitting the experimental release data values with the Ritger–Peppas equation are tabulated in Table 1. The NCLM and CLM nanocarriers without oxidation stimulus depicted *n* values of 0.34 and 0.31, respectively, both presenting a pseudo-Fickian diffusion mechanism. When the level of oxidation stimulus increased from 25 to 50 mM H_2_O_2_, the *n* values determined from the release profiles during the initial few hours were 0.58 to 0.65, suggesting an anomalous transport process. Under the above scenario, the micellar nanocarriers underwent a rapid structure disintegration due to the oxidation-triggered breakage of active Se-Se crosslinking sites, leading to an anomalous transport process comparable to diffusion accelerating the release of entrapped drugs.

### 2.8. Cytotoxicities of Diselenide-Crosslinked Micelles (CLMs)

**Figure 7:**
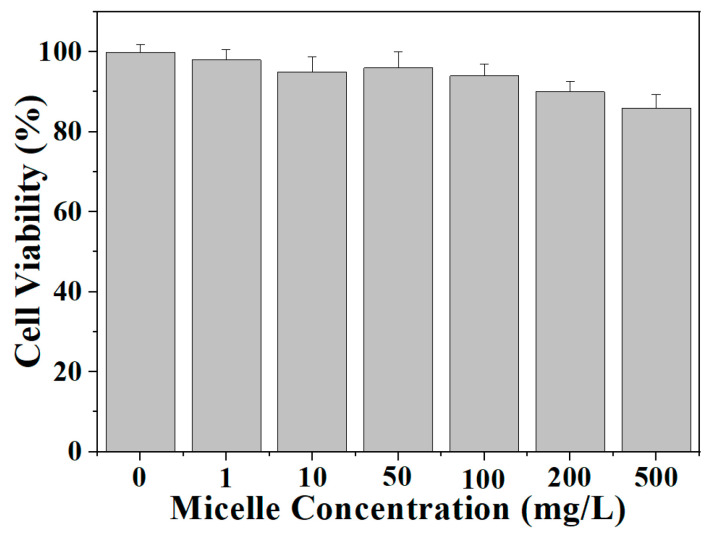
In vitro cytotoxicities of blank CLMs against HeLa cells after 48 h of incubation. Data are presented as mean ± SD (*n* = 3).

The cytotoxicities of the diselenide-crosslinked micelles were investigated using HeLa cells. As depicted in Figure 7, following a 24-h incubation period at concentrations ranging from 0 to 500 mg/L, only a slight reduction in cell viability was observed at higher concentrations, with percentages of 90% ± 2.6% and 86% ± 3.3% at 200 and 500 mg/L, respectively. These findings suggest that the diselenide-crosslinked micelles possessed satisfactory biocompatibility, allowing their safe application as nanocarriers in drug delivery systems.

### 2.9. Cellular Uptake of DOX-Loaded Micelles

**Figure 8:**
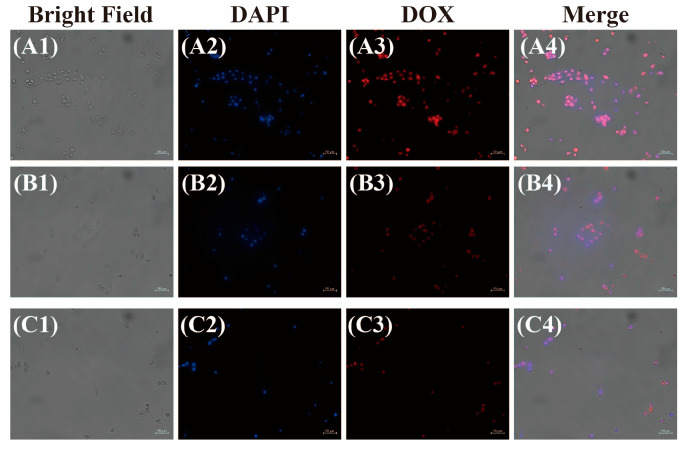
Fluorescence microscopy images of B16-F10 cells incubated with free DOX (**A1**–**A4**) and DOX-loaded NCLMs (**B1**–**B4**) and CLMs (**C1**–**C4**) for 4 h (Scale bar: 50 μm).

The cellular uptake behavior of the DOX-loaded micelles was further investigated via fluorescence microscopy in B16-F10 cells. Figure 8 shows the fluorescence microscopy images of the B16-F10 cells incubated for 4 h with the free DOX and DOX-loaded NCLMs and CLMs. It was found that the DOX-loaded NCLMs and CLMs displayed a similar fluorescence intensity of DOX in the B16-F10 cells. In contrast, the cells incubated with the free DOX displayed relatively higher fluorescence intensity than that of the DOX-loaded NCLMs and CLMs. This phenomenon can be attributed to two main factors. On the one hand, small-molecule chemotherapeutic drug DOX essentially enters the cell through diffusion, while most micelles are absorbed by the cell through endocytosis [53,54]. Therefore, the free DOX can penetrate the cell more effectively than the loaded DOX. On the other hand, the relatively weaker fluorescence intensity of the NCLMs and CLMs may also result from the self-quenching of DOX inside the micelles [55]. Furthermore, it is noteworthy that the red fluorescence from DOX was observed nearly completely overlapping with the blue cell nucleus, suggesting that the action site of this anti-tumor drug was the cell nucleus. DOX could be released from polymer micelles and penetrate the cell nucleus of the B16-F10 cells through free diffusion. These observations may indicate that the DOX-loaded CLMs can be efficiently internalized to release DOX inside the cells, facilitating the accumulation of anti-tumor drugs within cells and enhancing the bioavailability of the drugs. 

## 3. Materials and Methods

### 3.1. Materials

Selenium powder (200 mesh, 99.9%), sodium borohydride (NaBH_4_, 98%), 2,2-bis(bromomethyl)-1,3-propanediol (BMP, ≥98.0%), and doxorubicin hydrochloride (DOX, 98%) were obtained from Aladdin chemicals. Methoxy poly(ethylene glycol) (MPEG~2000), dibutyltin dilaurate (DBTDL, 97.5%), and pyrene (98%) were bought from J&K Chemicals (Beijing, China). Isophorone diisocyanate (IPDI, 99.5%) was obtained from Wanhua Chemical Co., Ltd. (Yantai, China). MPEG was pre-treated by dehydration under vacuum at 80 °C for 12 h before use. All the other reagents were supplied by Energy Chemical (Shanghai, China) and utilized without further treatment.

### 3.2. Instrumentation

The ^1^H and ^13^C NMR spectra analyses were carried out with a Bruker AV 400 spectrometer (Bruker, Bremen, Germany). The molecular weight and distribution of the samples were run on a Waters 1515-2414 gel permeation chromatography (GPC, Waters, Milford, MA, USA) apparatus with THF as the eluent and polystyrene (PS) standards. A PerkinElmer Lambda 750 spectrometer (PerkinElmer, Waltham, MA, USA) was used for the UV-vis spectroscopic analyses. The Fourier transform infrared (FT-IR) spectra were measured with a ThermoFisher Nicolet 5700 spectrophotometer (ThermoFisher, Waltham, MA, USA) using the traditional KBr pellet. The fluorescence spectroscopy analysis was performed with a PerkinElmer LS50 instrument (PerkinElmer Inc., Waltham, MA, USA). For the transmission electron microscopy (TEM) analyses, the samples were prepared by applying a drop-coating technique onto carbon-coated copper grids, followed by staining with a 2% solution of phosphotungstic acid and then recorded by a JEOL JEM-2100F TEM (JEOL Ltd., Tokyo, Japan). The sizes and distributions of the samples were analyzed at 25 °C using a Malvern Zetasizer Nano ZS instrument (Malvern Instrument Ltd., Malvern, UK). The differential scanning calorimetry (DSC) analyses of the lyophilized samples were conducted on a NETZSCH DSC/TG thermal analyzer (STA 449 F3 Jupiter, Netzsch, Selb, Germany) at a heating rate of 10 K min^−1^ under the N_2_ flow. Images of cellular fluorescence microscopy were captured using a Zeiss Inverted Axio Observer 7 Microscope (Carl Zeiss, Jena, Germany).

### 3.3. Synthesis of the Functional Monomer DiSe

The functional diselenolane diol, 4,4-bis(bishydroxymethyl)-1,2-diselenolane (DiSe), containing cyclic diselenide group, was synthesized with minor modifications based on the methodology described in the literature [48], as depicted in Scheme 2. Briefly, NaBH_4_ (1.0 g, 26.4 mmol) was dissolved in 30 mL of distilled water in an ice bath. Then, selenium powder (1.0 g, 12.6 mmol) was added and reacted under vigorous stirring for 10 min to give a colorless solution. Subsequently, a second addition of selenium powder with equal weight was made, then heated to 100 °C and kept under stirring for 30 min to give a brownish-red solution of sodium diselenide (Na_2_Se_2_). Then, a 20 mL ethanol solution of 2,2-bis (bromomethyl)-1,3-propanediol (BMP, 3.93 g, 15.0 mmol) was introduced into the Na_2_Se_2_ solution and reacted under stirring at 40 °C for 5 h. Upon filtration, the filtrate was further extracted using ethyl acetate and dehydrated using MgSO_4_. After purification by column chromatography and recrystallization twice from ethyl acetate, the final product DiSe was obtained as dark red needle-like crystals (28% yield). ^1^H NMR (400 MHz, CDCl_3_) δ ppm: 2.86 (s, 4H, -CH_2_SeSeCH_2_-), 3.87 (s, 4H, C(CH_2_OH)_2_). ^13^C NMR (400 MHz, CDCl_3_) δ ppm: 14.9 (SeCH_2_-), 49.0 (C(CH_2_OH)_2_), 68.9 (CH_2_OH).

### 3.4. Synthesis of MPEG-b-PUSe-b-MPEG Triblock Copolymers

The MPEG-*b*-PUSe-*b*-MPEG copolymers were synthesized through a polycondensation reaction of DiSe and IPDI, and terminated by MPEG, as shown in Scheme 2. Typically, DiSe (3 mmol, 0.78 g) and a catalytic quantity of DBTDL were dissolved in 25 mL of the anhydrous THF solution under the N_2_ flow. Then, a certain amount of IPDI (3.3 mmol, 0.73 g) was added and reacted at 50 °C under an N_2_ atmosphere for 2 h. Subsequently, MPEG (0.20 mmol, 0.40 g) was added and allowed to react for 12 h under stirring. Then, the resulting solution was precipitated with a large excess of diethyl ether. After three repeated precipitation–dissolution cycles, the purified product was finally dehydrated under vacuum to give a light brown powder with 86% yield. ^1^H NMR (400 MHz, CDCl_3_) δ ppm: 0.85-1.52 (C-(CH_3_)_2_, C-CH_3_ and -CH_2_- of IPDI), 3.24 (-CH_2_SeSeCH_2_-), 3.32 (CH_3_O- of MPEG), 3.95 (-O-CH_2_-C-CH_2_-O-), and 3.45-3.54 (-CH_2_CH_2_O- of MPEG). FT-IR (KBr, cm^−1^): 3365, 2951, 2920, 1643, 1560, 1464, 1110, and 774.

### 3.5. Preparation of Polymeric Micelles and Determination of Critical Micelle Concentration (CMC)

The polymeric micelles were prepared employing the dissolution and dialysis method. Typically, the triblock copolymer MPEG-*b*-PUSe-*b*-MPEG (150 mg) was dissolved in 5 mL of THF, then dropwise added into 100 mL distilled water, followed by dialysis against distilled water at room temperature for 24 h. Then, a homogeneous micellar solution was achieved and filtrated by 0.45 μm pore-size membranes for further application. The particle size, distribution, and morphology were examined using dynamic light scattering (Zetasizer Nano ZS, Malvern Instruments, Malvern, UK) and TEM (JEM-2100F, JEOL Ltd., Tokyo, Japan).

The CMC of the copolymer MPEG-*b*-PUSe-*b*-MPEG was determined via the pyrene probe approach. By fixing the final concentration of the pyrene probe at 1 × 10^−6^ mol/L, a series of concentration gradients of the copolymer ranging from 1 × 10^−5^ to 1 mg/mL were added and mixed thoroughly with pyrene overnight. The fluorescence intensities of the resulting solutions were recorded by a PerkinElmer LS50 fluorophotometer (PerkinElmer Inc., Waltham, MA, USA) with an excitation wavelength (λ_ex_) of 335 nm and emission wavelength of 373 nm (λ_ex1_) and 384 nm (λ_ex2_). The CMC values were determined from the inflection point of the curve plotted as the intensity ratio (I_384_/I_373_) versus polymer concentration.

### 3.6. Preparation of Diselenide Core-Crosslinked Micelles

The diselenide core-crosslinked MPEG-*b*-PUSe-*b*-MPEG micelles were prepared by visible-light-induced Se-Se bond-exchange reaction. A 20~30 W daylight desk lamp was used as the visible light source. The NCLMs (concentration: 0.5 mg/mL) were prepared via the above direct dissolution approach prior to use. Then, the diselenide-crosslinked micelles were achieved by subsequent irradiation under the desk lamp at a distance of 10 cm at room temperature for 30 min. DLS and TEM analyses were also carried out to determine the particle size and morphology after the visible light irradiation.

### 3.7. Structural Stability of Micelles

The structural stability experiments of the non-crosslinked and core-crosslinked micelles were tested using DMF (a good solvent for the as-prepared amphiphilic block polyurethanes) and pH~7.4 PBS solution (simulated physiological environment) as the dilution media. In brief, 5 mL of the prepared non-crosslinked or core-crosslinked micelles were diluted by adding different volumes of DMF solvent (or pH~7.4 PBS solution) and incubated overnight at room temperature for further characterization. Then, the colloidal properties of the above micelles were investigated using DLS and TEM to record the scattering light intensity, size distribution, and micromorphology.

### 3.8. ROS-Triggered Disassembly of Diselenide-Crosslinked Micelles

The ROS-induced disassembly behavior of the diselenide-crosslinked micelles (concentration: 0.5 mg/mL) was investigated using DLS and TEM. Typically, 10 mL of the as-prepared crosslinked micelles were sealed with a dialysis bag (cutoff 3500 Da) and placed into 100 mL of the 50 mM H_2_O_2_ solution. After incubation at room temperature for 24 h, the particle size and morphology variations were investigated using the DLS and TEM analyses.

### 3.9. In Vitro Drug Loading and Release

The model drug DOX was selected to investigate the drug loading and release properties. The DOX-loaded micelles were prepared using the typical dialysis method. Typically, DOX (50 mg), equivalent triethylamine, and MPEG-*b*-PUSe-*b*-MPEG (150 mg) were mixed thoroughly in 5 mL of THF. Then, a thin film of the DOX-containing polymer was prepared by evaporation at room temperature. Subsequently, 100 mL of distilled water was added, followed by sonication for 15 min and mild stirring in the dark for 12 h at room temperature. After subsequent dialysis against distilled water in the dark for 24 h, the DOX-loaded NCLMs were obtained with a micelle concentration of 1 mg/mL and passed through a 0.45 μm pore-size syringe membrane before use. Upon further treatment by visible light irradiation for 30 min, the DOX-loaded diselenide-crosslinked micelles were prepared. The DLC and DLE of the as-prepared micelles were determined by a UV/vis spectrometer (UV-2600, Shimadzu, Kyoto, Japan). Typically, the above-prepared DOX-loaded micelle solutions were lyophilized and redispersed in the DMF solvent. The absorbance of these solutions was recorded at 482 nm and quantitatively analyzed by comparison with a standard curve. Then, the DLC and DLE values were calculated as follows:

DLC = (weight of loaded DOX/weight of DOX-loaded micelles) × 100%

DLE = (weight of loaded DOX/weight of DOX in feed) × 100%

The stimuli-triggered release behaviors of the DOX-loaded micelles were conducted under oxidative conditions. The H_2_O_2_-containing PBS (pH 7.4) solutions with different concentrations (0, 25, or 50 mM) were adopted as the release medium. A total of 10 mL of the DOX-loaded micelles sealed by a dialysis bag was immersed into the release medium and incubated at 37 °C under mild shaking (130 rpm). In total, 3 mL of the above release medium was collected at regular intervals to examine the absorbance at 482 nm, and an equal volume of the fresh release medium was added. The corresponding DOX release amount was determined depending on the standard curve. All the analyses were conducted in triplicate.

### 3.10. MTT Assay

The cell viability was tested using an MTT assay. Briefly, HeLa cells were seeded in 96-well plates with a density of 5000 cells per well in 200 μL Dulbecco’s Modified Eagle Medium (DMEM) with 10% fetal bovine serum (FBS), and incubated at 37 °C with 5% CO_2_ overnight. Subsequently, the medium was removed and replaced by fresh medium with different concentrations of the MPEG-*b*-PUSe-*b*-MPEG polymers (0~500 mg/L). After incubation for 72 h, 20 μL of 5 mg/mL 3-(4,5-Dimethyl-2-thiazolyl)-2,5-diphenyl-2H-tetrazolium bromide (MTT, Sigma-Aldrich, Shanghai, China) PBS solution was added into each well and incubated at 37 °C for another 4 h. Then, the medium was removed and 100 μL DMSO was added to dissolve the MTT-formazan crystals. The absorbance of each well was recorded at 570 nm by a microplate reader (EnSpire, PerkinElmer). Cell viability was determined by the following equation: Cell viability (%) = (A_treated_ − A_0_)/(A_control_ − A_0_) × 100%, where A_treated_, A_control_, and A_0_ represent the absorbance of the sample well, control well, and blank well at 570 nm, respectively.

### 3.11. Cellular Uptake Measurements

Mouse skin melanoma B16-F10 cells were used to track the cellular uptake behavior of the DOX-loaded micelles. The B16-F10 cells were seeded in a 6-well plate (5 × 10^5^ cells per well) in DMEM containing 10% FBS, 1% penicillin, and streptomycin, and incubated in an incubator with 5% CO_2_ at a constant temperature of 37 °C for 24 h. Subsequently, the culture medium was aspirated and replaced with PBS solutions containing the free DOX or DOX-loaded micelles at a concentration of 10 μg/mL (DOX dosage). After further incubation for 4 h, the culture medium was removed, and the cells were washed three times using PBS. The cells were then fixed using 4% paraformaldehyde for 10 min and washed thrice using PBS. The subsequent staining of the cell nuclei was performed using a DAPI staining solution for 5 min, and the cell nuclei were further washed three times using PBS. The fluorescence microscope images of the as-treated cells were captured using an inverted fluorescence microscope (Zeiss Axio Observer 7, Carl Zeiss, Jena, Germany).

## 4. Conclusions

A novel type of diselenide-crosslinked polymeric micelles was easily synthesized via the visible-light-induced diselenide metathesis reaction. The DLS and TEM analyses showed that the size of the micelles was decreased to 90 nm after the visible-light-induced crosslinking process. These diselenide-crosslinked polymeric micelles showed enhanced colloidal stability and could efficiently encapsulate model drugs like DOX with a higher DLC of 20.7% ± 1.9% and DLE of 72.6% ± 4.6%. Additionally, they revealed minimal drug leakage and triggered drug release behaviors upon exposure to ROS. Moreover, these diselenide-crosslinked nanocarriers also had good biocompatibility and efficient cellular uptake behavior. Considering their ease of preparation, enhanced colloidal stability, good biocompatibility, efficient cellular uptake, and oxidation-triggered controlled drug release properties, these diselenide-crosslinked micellar nanocarriers could be a promising candidate for controlled drug delivery systems.

## Figures and Tables

**Table 1:** Kinetic constant (*k*), release exponent (*n*), correlation coefficient (*R*^2^), and the corresponding release mechanism of the NCLM and CLM nanocarriers under different circumstances.

Nanocarrier	Release Medium	*k*	*n*	*R* ^2^	Release Mechanism
NCLMs	0 mM H_2_O_2_	12.25	0.34	0.93	pseudo-Fickian
CLMs	0 mM H_2_O_2_	5.74	0.31	0.98	pseudo-Fickian
CLMs	25 mM H_2_O_2_	12.00	0.58	0.99	Anomalous transport
CLMs	50 mM H_2_O_2_	16.70	0.65	0.99	Anomalous transport

## Data Availability

The data presented in this study are available upon request from the corresponding author.

## Supplementary Materials

The following supporting information can be downloaded at: https://www.mdpi.com/article/10.3390/molecules29163970/s1, Figure S1: ^13^C NMR spectrum of the functional monomer diselenolane diol; Figure S2: FT-IR (A) and ^1^H NMR (B) spectra of PUSe prepolymers; Figure S3: I_384_/I_373_ intensity ratios from pyrene excitation spectra as a function of concentration of MPEGPUSe-MPEG in aqueous solution; Figure S4: ^1^H NMR spectra of diselenolane diol before (up) and after (down) Vis-irradiation; Figure S5: Size distributions of non-crosslinked (NCLMs) and crosslinked micelles (CLMs) against 100-fold dilution by pH 7.4 PBS solution; Figure S6: Size distributions of DOX-loaded non-crosslinked (DOX-NCLMs) and crosslinked micelles (DOX-CLMs) before and after incubation in pH 7.4 PBS solution for 40 h.
