# Supplementary material for: Visible-Light-Induced Diselenide-Crosslinked Polymeric Micelles for ROS-Triggered Drug Delivery
Source: Molecules. 2024 Aug 22;29(16):3970. doi: 10.3390/molecules29163970 (PMC11357037; doi:10.3390/molecules29163970)
Supplement: Supplementary file 1 [file molecules-29-03970-s001.zip › molecules-3036732-supplementary.pdf]

Supporting Information for:

Visible light-induced diselenide-crosslinked polymeric micelles for ROS-triggered drug delivery

Xinfeng Cheng [1,*], Huixian Li[1], Xiaomeng Sun[1], Tianxu Xu[1], Zhenzhen Guo[1], Xianchao Du[1], Shuai Li[1], Xuyang Li[1], Xiaojing Xing[1], and Dongfang Qiu [1,*]

[1]College of Chemistry and Pharmaceutical Engineering, Nanyang Normal University, Nanyang 473061, P.R. China

[*]Correspondence: x.f.cheng@nynu.edu.cn (X.F.C.); qiudf2008@nynu.edu.cn (D.F.Q.).

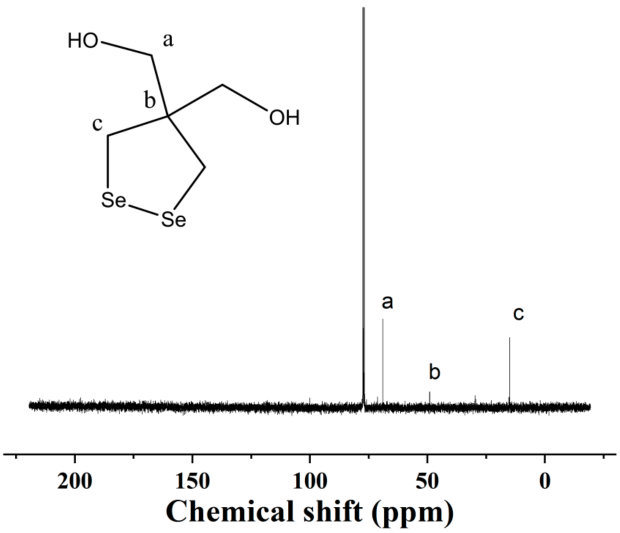

Figure S1. ^{13}C NMR spectrum of the functional monomer diselenolane diol.

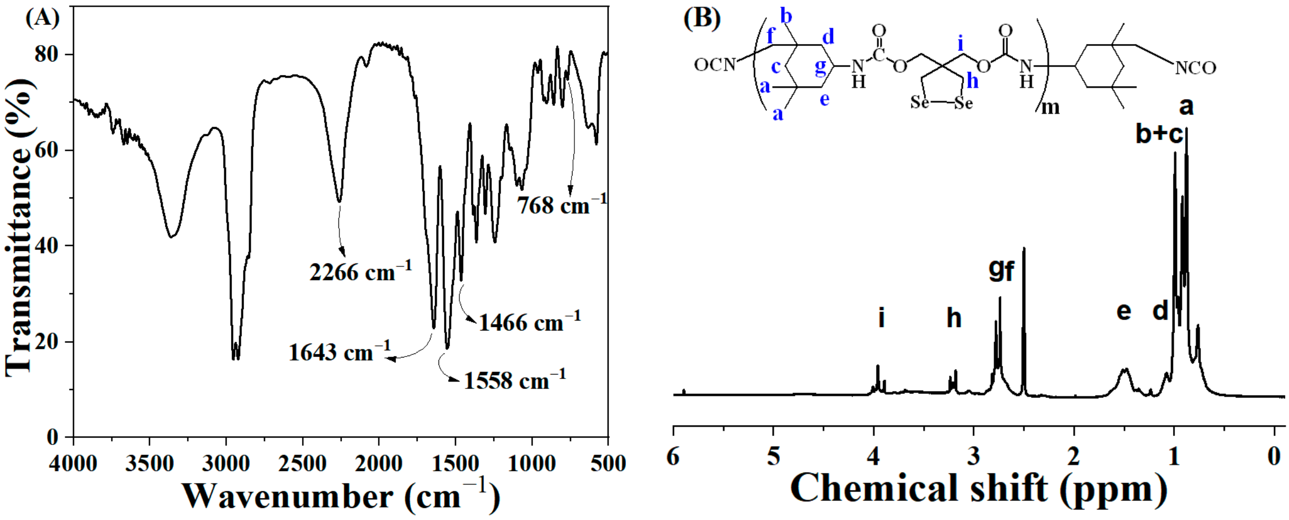

Figure S2. FT-IR (A) and ^{1}H NMR (B) spectra of PUSe prepolymers.

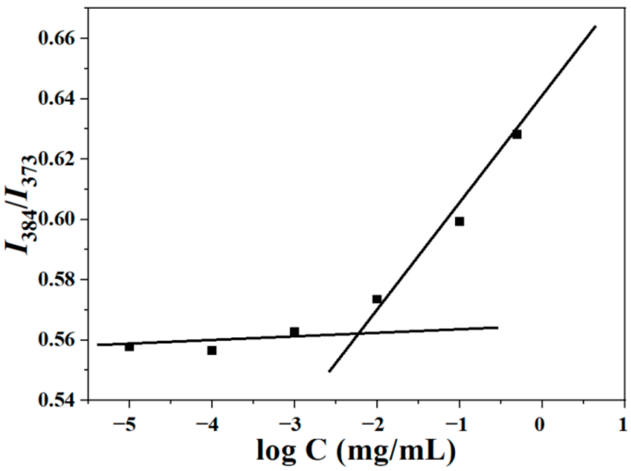

Figure S3. I_{384}/I_{373} intensity ratios from pyrene excitation spectra as a function of concentration of MPEGPUSe-MPEG in aqueous solution.

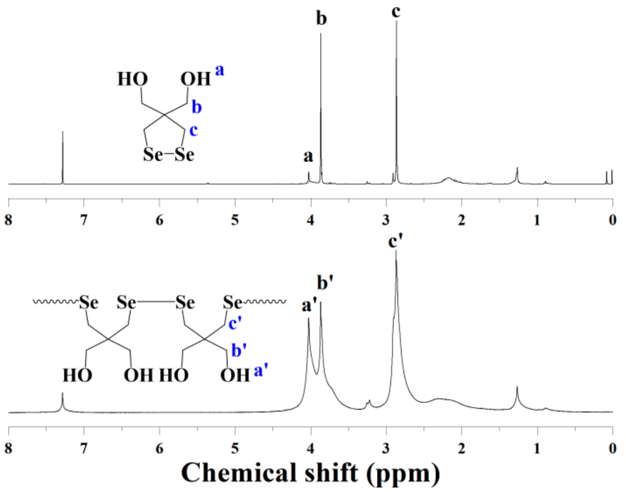

Figure S4. ^{1}H NMR spectra of diselenolane diol before (up) and after (down) Vis irradiation.

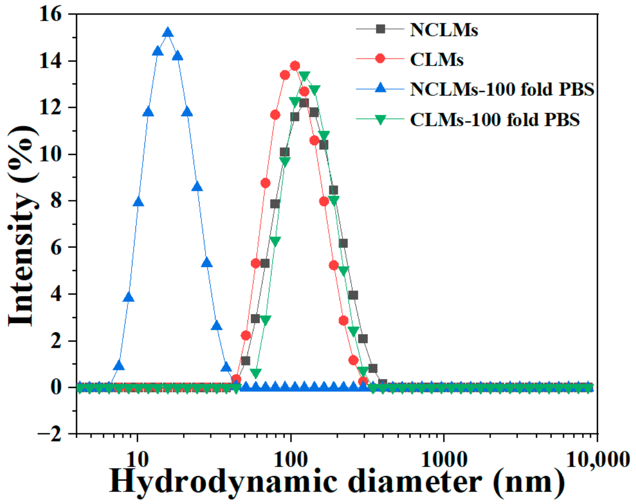

Figure S5. Size distributions of none-crosslinked (NCLMs) and crosslinked micelles (CLMs) against 100-fold dilution by pH 7.4 PBS solution.

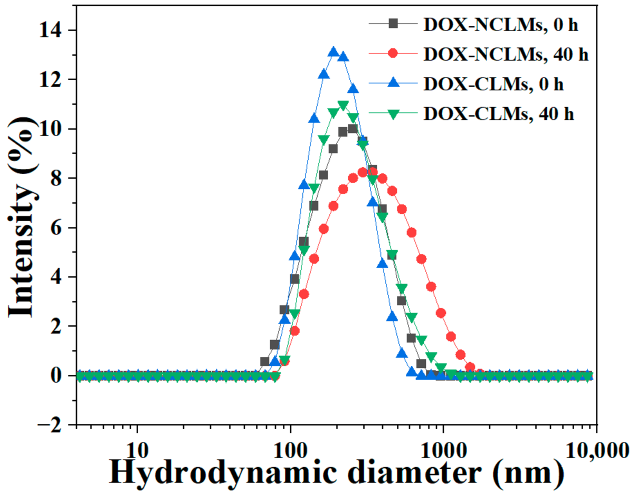

Figure S6. Size distributions of DOX-loaded none-crosslinked (DOX-NCLMs) and crosslinked micelles (DOX-CLMs) before and after incubation in pH 7.4 PBS solution for 40 h.
